# Cholesterol, Atherosclerosis, and APOE in Vascular Contributions to Cognitive Impairment and Dementia (VCID): Potential Mechanisms and Therapy

**DOI:** 10.3389/fnagi.2021.647990

**Published:** 2021-03-25

**Authors:** Michael Tran Duong, Ilya M. Nasrallah, David A. Wolk, Catherine C. Y. Chang, Ta-Yuan Chang

**Affiliations:** ^1^Perelman School of Medicine, University of Pennsylvania, Philadelphia, PA, United States; ^2^Geisel School of Medicine, Dartmouth College, Hanover, NH, United States

**Keywords:** cholesterol, atherosclerosis, APOE, vascular dementia, inflammation, glia, macrophage

## Abstract

Vascular contributions to cognitive impairment and dementia (VCID) are a common cause of cognitive decline, yet limited therapies exist. This cerebrovascular disease results in neurodegeneration *via* acute, chronic, local, and systemic mechanisms. The etiology of VCID is complex, with a significant impact from atherosclerosis. Risk factors including hypercholesterolemia and hypertension promote intracranial atherosclerotic disease and carotid artery stenosis (CAS), which disrupt cerebral blood flow and trigger ischemic strokes and VCID. Apolipoprotein E (APOE) is a cholesterol and phospholipid carrier present in plasma and various tissues. APOE is implicated in dyslipidemia and Alzheimer disease (AD); however, its connection with VCID is less understood. Few experimental models for VCID exist, so much of the present information has been drawn from clinical studies. Here, we review the literature with a focus on the clinical aspects of atherosclerotic cerebrovascular disease and build a working model for the pathogenesis of VCID. We describe potential intermediate steps in this model, linking cholesterol, atherosclerosis, and APOE with VCID. APOE4 is a minor isoform of APOE that promotes lipid dyshomeostasis in astrocytes and microglia, leading to chronic neuroinflammation. APOE4 disturbs lipid homeostasis in macrophages and smooth muscle cells, thus exacerbating systemic inflammation and promoting atherosclerotic plaque formation. Additionally, APOE4 may contribute to stromal activation of endothelial cells and pericytes that disturb the blood-brain barrier (BBB). These and other risk factors together lead to chronic inflammation, atherosclerosis, VCID, and neurodegeneration. Finally, we discuss potential cholesterol metabolism based approaches for future VCID treatment.

## Introduction

**Figure 1:**
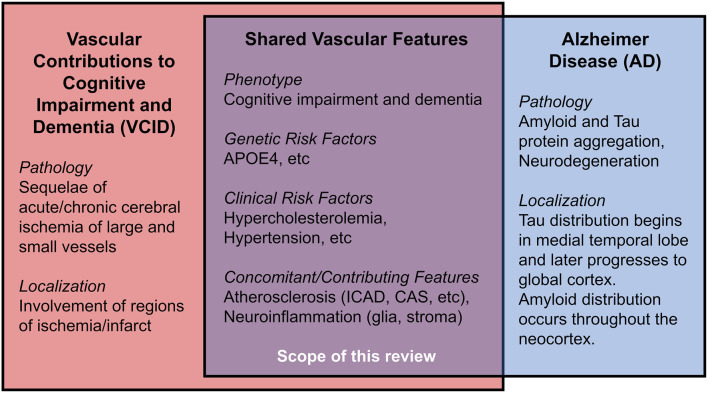
Shared features in Vascular Contributions to Cognitive Impairment and Dementia (VCID) and Alzheimer Disease (AD).

Vascular contributions to cognitive impairment and dementia (VCID) are defined by cognitive impairment secondary to acute and/or chronic cerebral ischemia and encompass the classical term, vascular dementia (Gorelick et al., 2011; Iadecola et al., 2019). VCID is the most common form of secondary neurodegeneration worldwide; one out of six people with dementia have VCID (van der Flier and Scheltens, 2005). The four subtypes of VCID are: (1) post-stroke, manifesting within 6 months after infarct; (2) subcortical ischemia, including small-vessel occlusion; (3) multi-infarct, including medium-to-large vessel disease; and (4) mixed, incorporating vascular and protein aggregate pathologies (Skrobot et al., 2018). The most common etiology of dementia is Alzheimer disease (AD), a form of cognitive impairment with amnestic-predominant phenotype and the presence of cerebral amyloid and tau protein aggregates (Jack et al., 2018). Compared to the distinct anatomical pattern of AD neurodegeneration originating within the medial temporal lobe, neurodegeneration linked to VCID occurs secondary to focal or global insufficiency of cerebral blood supply. Mixed dementia includes contributions from vascular and other pathologies (often AD) and comprises about 20% of all dementia cases (van der Flier and Scheltens, 2005; Suemoto et al., 2017). Several VCID animal models exist, though they are limited in their capacity to comprehensively recapitulate mixed pathologies/phenotypes in humans (Gooch and Wilcock, 2016). This review article focuses on the clinical aspects of certain risk factors for VCID, i.e., cholesterol, atherosclerosis, and APOE4, as well as the relationship of these risk factors for AD. We hope to offer new insight into disease mechanisms and future therapies (Figure 1).

In 1894, Otto Binswanger provided the first clinicopathologic description of VCID. His patients demonstrated focal neurodegeneration where cerebral arteries displayed “extensive atherosclerotic changes,” “fatty degeneration,” and “thickening of inner and middle vascular membranes.” Strikingly, he noted a “primary proliferation of the glial parts” surrounding lipid-filled vessels (Binswanger, 1894; Blass et al., 1991). Thus, VCID is strongly associated with cerebral **atherosclerosis**, the chronic dysfunction of lipid homeostasis, and local inflammation caused by the accumulation of **cholesterol**, **cholesteryl esters (CEs)**, other lipids, and activated stromal cells, including lipid-laden foamy macrophages, endothelial, and smooth muscle cells of vessel walls (Glass and Witztum, 2001; Hansson et al., 2006). Since perivascular astrocytes and pericytes modulate local blood vessel diameter to reflect real-time neuronal activity through “neurovascular coupling” (Attwell et al., 2010), it is reasonable to consider how activated glia and pericytes respond and contribute to intracranial atherosclerosis and ischemia (Binswanger, 1894; MacVicar and Newman, 2015; Price et al., 2018; Fernandez et al., 2019), particularly regarding lipid regulation (Koizumi et al., 2018). Atherosclerosis causes vessel stenosis and occlusion, thereby reducing cerebral blood flow. VCID is often associated with hypercholesterolemia, wherein elevated serum cholesterol promotes a cascade of cerebrovascular cholesterol deposition, inflammation, ischemia, neuronal injury, and cognitive impairment (Solomon et al., 2009; Appleton et al., 2017). We frame atherosclerosis as a condition associated with cerebrovascular lipid deposition and disease of large, medium, and small vessels, including arteriosclerosis and lipohyalinosis. While this review focuses on atherosclerotic influences on VCID, it is important to note that additional vascular etiologies contribute to VCID, including ischemia due to thromboembolism (cardiogenic sources, coagulopathy), chronic hypoperfusion, hypertension, et cetera (Qiao et al., 2017). These factors are covered in several reviews (Gorelick et al., 2011; Santos et al., 2017; Wolters and Ikram, 2019).

**Apolipoprotein E (APOE)** is a lipid-carrier protein tightly linked to dementia (Strittmatter et al., 1993). The *APOE* ε4 allele (**APOE4**), a minor allele of the *APOE* gene, is associated with a higher risk for AD (>2-fold increased risk for heterozygotes, >9-fold risk for homozygotes) and an elevated risk for VCID (2-fold increased risk for heterozygotes, 3-fold risk for homozygotes) (Rasmussen et al., 2018). Generally, APOE4 is linked to early memory impairment (Caselli et al., 2007), limbic dysfunction (Wolk and Dickerson, 2010), white matter (WM) ischemia (Koizumi et al., 2018), aberrant lipid metabolism, and neuroinflammation (Liu et al., 2013; Rasmussen, 2016; Tzioras et al., 2019). We then explore APOE4 in atherosclerosis and VCID.

## Working Model of Atherosclerosis, Apoe, and Vcid

Here, we synthesize clinical data on cholesterol, atherosclerosis, APOE, and VCID into a working model; we posit that atherosclerosis and APOE4 promote reoccurring occlusion and ischemia that eventually lead to VCID. Then, we discuss potential intermediate steps involved, based on emerging research.

### Atherosclerosis Is Associated With VCID and AD

Atherosclerosis can be divided into several categories: cerebral atherosclerosis that affects distal microscopic vessels, intracranial atherosclerotic disease (ICAD) that affects cerebral arteries, and large-vessel disease such as carotid artery stenosis (CAS) affecting carotid arteries supplying the brain (Box 1, Supplementary Figure 1). Myriad clinical data support the link between atherosclerosis and VCID as well as AD. For instance, cerebral autopsies (Beach et al., 2017) and Doppler ultrasound investigations of internal carotid arteries (Hofman et al., 1997) reveal vessel stenosis is more frequently observed in VCID and AD than in normal cognition, with a stronger association between VCID and atherosclerosis than AD with atherosclerosis. Additional studies support the association of atherosclerosis with dementia (Dolan et al., 2010; Dearborn et al., 2017), and with AD particularly (Roher et al., 2011; Yarchoan et al., 2012). In *APOE4* carriers, ICAD/CAS is associated with greater cognitive decline (Haan et al., 1999).

Box 1 **Glossary.**

**Carotid Artery Stenosis (CAS)**: Narrowing of intracranial, extracranial, and/or common carotid arteries that supply the head, neck, and Circle of Willis of the brain.

**Circle of Willis (CoW)**: A circular network of cerebral arteries fed from carotid and vertebral arteries that deliver blood to the brain. ***Intracranial Atherosclerotic Disease (ICAD)***: Deposition of lipids, debris, and inflammatory cells in the walls of arteries in the skull that supply the brain. This condition is closely associated with VCID.

**Ischemia**: Loss of blood and oxygen supply to the tissue that can be chronic (atherosclerosis), or acute (sudden occlusion).

**Occlusion**: Blockage of a vessel leading to ischemia. In the brain, arterial occlusion causes a stroke, the occurrence of sudden neurologic deficit(s) due to inadequate vascular supply.

**Stromal Activation**: Proliferation and reaction of non-neuronal cells responding to and often exacerbating the neuronal injury. This includes activation of astrocytes, microglia, oligodendrocytes, vessel endothelial cells, pericytes, smooth muscle cells, macrophages, and other immune cells.

**Vascular Contributions to Cognitive Impairment and Dementia (VCID)**: Cognitive impairment or dementia due to acute and/or chronic ischemia, secondary to a stroke, intracranial atherosclerosis, carotid artery stenosis, et cetera or a combination of vascular disease, Alzheimer neuropathologic changes (amyloid/tau), and/or other pathologies.

### Relationships Between Atherosclerosis, Amyloid, and Tau

Amyloid and tau pathologies are the two distinct criteria for AD; they can also be present in VCID. Patients with suspected VCID and appreciable amyloid/tau biomarkers may have mixed dementia (Skrobot et al., 2018). Alterations in lipid deposition and cholesterol metabolism may modulate amyloid and/or tau pathology (Pappolla et al., 2002). Recent evidence from *in vivo* and *ex vivo* studies suggests that vascular lipid dysregulation and atherosclerosis may be independent dementia risk factors, in addition to their effects on amyloid/tau pathology. Several studies support this view: autopsies indicate ICAD does not correlate with amyloid/tau in either aging patients (Dolan et al., 2010), or in AD cohorts (Kosunen et al., 1995). ICAD severity on magnetic resonance angiography does not correlate cross-sectionally or spatially with *in vivo* cerebral amyloid burden based on positron emission tomography imaging (Gottesman et al., 2020). ICAD and AD elicit similar proteomic alterations in human dorsolateral prefrontal cortex glia and oligodendrocytes (Wingo et al., 2020). Interestingly, ICAD but neither amyloid nor tau pathology was associated with neurodegeneration, as measured by neurofilament light elevation (Iadecola, 2020; Wingo et al., 2020). Thus, amyloid may exert vascular changes through distinct paths from those caused by hypercholesterolemia and ICAD. Cerebral amyloid angiopathy (CAA), the deposition of amyloid aggregates in cerebral vessels, is associated with AD and VCID; moreover, both APOE4 and ICAD are associated with CAA and neurodegeneration (Premkumar et al., 1996; Tian et al., 2004; Yarchoan et al., 2012). While the *APOE*ε2 allele (APOE2) is associated with lower AD risk, it raises CAA risk (Nelson et al., 2013), demonstrating a complex interplay between cholesterol, APOE, and parenchymal vs vascular amyloid. *These data suggest vascular atherosclerosis and AD pathologies are two dissociable yet interacting contributions to neurodegeneration and cognitive decline*.

### APOE4 Is Associated With VCID and AD by Promoting Atherosclerosis

There is a clear association between *APOE* genotype and elevated VCID risk, validated through population studies (Slooter et al., 1997; Chang et al., 2010; Beach et al., 2017; Rasmussen et al., 2018; Pendlebury et al., 2020) and meta-analyses (McCarron et al., 1999). Studies investigating APOE4 in dementia after acute ischemic infarcts suggest that APOE4 impedes stroke recovery and promotes post-stroke VCID (Slooter et al., 1997; Pendlebury et al., 2020; Montagne et al., 2020b). Likewise, *APOE4* carriers have an elevated risk of severe cardiac, extracranial and intracranial atherosclerosis (Mahley and Rall, 2000; Bennet et al., 2007; Granér et al., 2008). These findings are corroborated by post-mortem analyses of atherosclerosis in the Circle of Willis (CoW; Box 1; Kosunen et al., 1995; Abboud et al., 2008), and by *in vivo* internal carotid artery imaging studies (Terry et al., 1996; Cattin et al., 1997; Haan et al., 1999; Elosua et al., 2004; Volcik et al., 2006). A meta-analysis of 490 case-control studies also supports the impact of APOE4 on ICAD risk (Wei et al., 2017). Notably, CAS risk may be greater in middle-aged asymptomatic *APOE4* carriers (Cattin et al., 1997), and ICAD may be associated more with male *APOE4* carriers (Elosua et al., 2004; Abboud et al., 2008). The latter finding is distinct from AD pathology, where APOE4 is more strongly linked to AD risk in females (Neu et al., 2017). Overall, the association between APOE and cerebrovascular disease is affected by age and sex (Liu et al., 2013; Beach et al., 2017; Hohnman et al., 2018; Lamar et al., 2019). It should be noted that other studies suggest that APOE4 may not be associated with CoW ICAD severity (Premkumar et al., 1996; Yarchoan et al., 2012; Beach et al., 2017). However, on balance, a substantial plurality of studies strongly indicates a close association exists between APOE4 and ICAD.

## Refining The Model: Intermediate Steps

The mechanisms leading from APOE4 to atherosclerosis to VCID are complex. Here, we consider candidate steps that may act as mediators, including serum/brain cholesterol, neurological/systemic inflammation, blood-brain permeability, and vascular aging. This requires the integration of clinical evidence with insight from animal and *in vitro* studies. It is important to note that additional vascular risk factors such as hypertension and smoking also influence dementia risk *via* multiple mechanisms to affect ICAD, stroke, and WM ischemia (Skoog et al., 1998; Kivipelto et al., 2001; Qiao et al., 2017; Koizumi et al., 2018; Nasrallah et al., 2019).

### Hypercholesterolemia, APOE4, and Dementia

Hypercholesterolemia is associated with atherosclerotic cardiovascular and cerebrovascular disease (Duncan et al., 2019). Indeed, cohort studies and meta-analyses reveal that hypercholesterolemia at various times in the lifespan significantly increases the risk for ICAD (Ritz et al., 2014) and VCID (Moroney et al., 1999; Reitz et al., 2004). Elevated serum cholesterol may also promote AD, likely through mixed pathologies (Kivipelto et al., 2001; Solomon et al., 2009; Anstey et al., 2017; Wingo et al., 2019; Zhou et al., 2020). Mid-life cholesterol elevation is significantly associated with AD and trended towards elevated risk in VCID (Solomon et al., 2009). Furthermore, patients with familial hypercholesterolemia have a higher risk of mild cognitive impairment (Zambón et al., 2010). It is worth noting that most, but not all studies (Slooter et al., 1999; Mielke et al., 2010), show that hypercholesterolemia significantly raises dementia risk. Atherosclerosis risk and hypercholesterolemia are linked by mechanisms that involve APOE. APOE is present in plasma lipid particles as well as various cell types in the body and brain, as an essential lipid-carrier. It binds to low-density lipoprotein (LDL) receptors and other related receptors (Herz, 2009; Liu et al., 2013). While APOE2 is associated with elevated very-low-density lipoprotein (VLDL) and lower dementia risk (Reiman et al., 2020), APOE4 is linked to higher LDL cholesterol (Beilby et al., 2003; Saito et al., 2004; Hall et al., 2006; Bennet et al., 2007) and increased dementia risk.

### Lipid Load in Astrocytes and Microglia, Neuroinflammation, and APOE4

The relationship between glia and lipid deposition traces to the original discoveries of VCID and AD, wherein Alzheimer observed that “many glial cells show adipose saccules” (Alzheimer, 1907; Stelzmann et al., 1995), and Binswanger noted the presence of rich “glial coating” of atherosclerotic vessels displaying “fatty degeneration” (Binswanger, 1894; Blass et al., 1991). Yet, only recently has local lipid deposition regained attention. Post-mortem lipidomic analyses of AD brains reveal deposits of a few selected lipid species, including cholesteryl esters (CEs), sphingomyelin, and ganglioside GM3. These species were enriched in AD-vulnerable regions (i.e., entorhinal and prefrontal cortex), but not cerebellum (Chan et al., 2012).

Astrocytes play essential roles in neuroinflammation and lipid deposition. Cumulatively, evidence indicates that astrocyte activation is linked to intracellular brain lipid accumulation, and APOE4 may exacerbate this response. For instance, human stem cell-derived APOE4 astrocytes display higher intracellular and extracellular cholesterol load, and compromised cholesterol efflux (Lin et al., 2018; Julia et al., 2019), as well as impaired endocytosis of lipids, amyloid, and other proteins, relative to astrocytes without APOE4 (Fernandez et al., 2019; Narayan et al., 2020). Similarly, cholesterol metabolism disruption was observed in human astrocytes expressing AD mutations, and these abnormalities were associated with tau hyperphosphorylation and neuronal toxicity (van der Kant et al., 2019). Lipid accumulation in AD astrocytes might be associated with aberrant acetate/acetyl-CoA metabolism, fatty-acid oxidation, and oxidative stress, which are markers for astrocyte activation (Wyss et al., 2011; Fernandez et al., 2019).

Microglia likely act as neuroinflammatory intermediaries between lipid overload and neurodegeneration. Lipid-laden microglia trigger oxidative stress and release proinflammatory cytokines (Marschallinger et al., 2020). Strikingly, numerous dementia-associated genes (i.e., *APOE*, *ABCA1*, *ABCA7, CLU*, *PLCγ2, TREM2*) are critical to lipid homeostasis and selectively expressed in microglia (Verheijen and Sleegers, 2018). Studies in mouse models demonstrate that deficiency of either *APOE* or *TREM2* impairs phagocytosis, abrogates clearance of myelin-derived lipids, and promotes CE buildup (Nugent et al., 2020). ABCA1 is a key mediator of cellular lipid efflux. Variants in *ABCA1* are implicated in cerebrovascular disease and AD (Nordestgaard et al., 2015). Further, *PLCγ2* and *TREM2* form a vital microglial lipid-sensing axis; genetic knockout of either gene in human stem cell-derived microglia models cause accumulation of cholesterol, CEs, and myelin-derived lipids, and perturbed inflammatory response (Andreone et al., 2020). APOE4 contributes to an inflammatory cascade of the neurovascular milieu (Lathe et al., 2014; Fernandez et al., 2019; Tzioras et al., 2019), likely in response to amyloid and lipid load. In APOE4 human microglia models, immunity/inflammation transcriptional pathways are dysregulated (Lin et al., 2018). Interestingly, in AD mouse models, *Apoe* expression occurs in the “late response” phase of microglial activation, possibly indicating a role for microglial *APOE* in chronic neuroinflammation and dementia (Keren-Shaul et al., 2017; Mathys et al., 2017).

### Macrophages in Atherosclerosis and Inflammation

Macrophages are innate immune phagocytes found in both systemic and brain tissue. Macrophages display phenotypic plasticity in gene expression and cell function. In mouse models and in human atherosclerotic plaques, macrophages can be categorized into several distinct categories, including resident, proinflammatory, and foamy macrophages (Cochain et al., 2018). The cholesterol-rich, lipid-laden foamy macrophages are hallmarks of the early stages of atherosclerosis (Glass and Witztum, 2001). Surprisingly, foamy macrophages are distinct from other proinflammatory macrophages and even share transcriptional similarities to activated smooth muscle cells in atherosclerotic lesions (Winkels et al., 2018; Zernecke et al., 2020). These foamy macrophages express high levels of *APOE* and *TREM2*, suggesting crucial roles for APOE and TREM2 in maintaining macrophage cholesterol homeostasis (Zernecke et al., 2020). These studies illustrate the diversity and complexity of responses to lipid deposition in various immune and stromal cells. Foam cells in atherosclerosis may be analogous to the aging, cholesterol-rich, lipid-laden glia in the brain; *APOE* and *TREM2* may play key roles in maintaining proper cholesterol homeostasis in the foamy microglia and astrocytes. Importantly, macrophages and glia regularly interact at key interfaces in glymphatic pathways, meningeal lymphatic vessels, and perivascular spaces (Louveau et al., 2017). Such crosstalk is integral to understanding inflammatory and vascular contributions to neurodegeneration.

### Commonalities Between VCID and AD: Cholesterol, APOE4, and Inflammation

**Figure 2:**
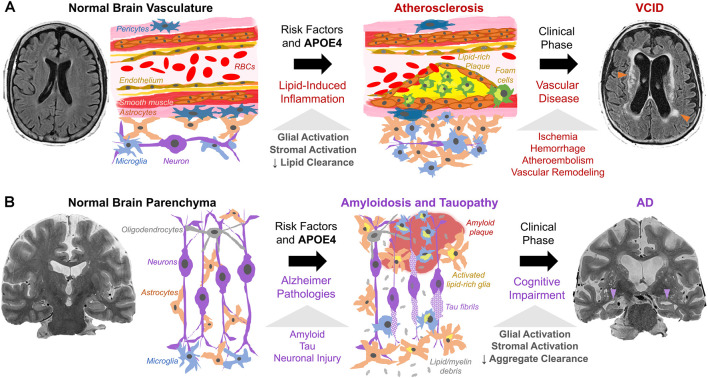
Working model of cholesterol, APOE4, and stromal activation in (**A**) VCID and (**B**) AD. (**A**) Risk factors (hypercholesterolemia, hypertension, smoking, APOE4) trigger atherosclerosis *via* cholesterol-rich plaques with local/systemic inflammation and stromal dysfunction (glia, macrophages, smooth muscle, endothelium, pericytes). Lipid-induced inflammation impairs lipid turnover and exacerbates plaque formation. This leads to ischemia (arrowheads) and dementia as detected by fluid-attenuated inversion recovery MRI. (**B**) Amyloid and tau pathology, with risk factors and APOE4, trigger neuronal injury. Subsequent proteinopathy-associated glial activation and lipid deposits (from glial proliferation and myelin debris) hinder amyloid/tau aggregate clearance and promote accumulation. This leads to aggregate spread and neurodegeneration (arrowheads) as seen on T2-weighted MRI.

Lipid deposition (both intracellular and extracellular) and chronic inflammation are shared between atherosclerosis, VCID, and AD. In VCID, cholesterol, CEs, and other lipids accumulate in macrophages and cerebral blood vessels and impair blood flow (Figure 2A). In AD, similar lipid species accumulate mainly in glia and impede clearance of amyloid/tau aggregates (Figure 2B). In VCID and AD, crosstalk of systemic and neuroinflammatory pathways leads to stromal dysfunction (Holmes et al., 2009; Tao et al., 2018). *Hence, lipid-associated inflammation alters glial, myeloid, and stromal cell interactions, hampers the turnover of accumulated lipid and protein aggregates, and promotes neurodegeneration*. It is important to determine how/when proposed inflammatory cascades occur and how/when lipid overload and APOE4 spur local/systemic inflammation and worsen ICAD, VCID, and AD. Together, cholesterol accumulation, subsequent neuroinflammation, and impaired phagocytosis may be common features across the neurodegenerative spectrum.

### Blood-Brain Barrier

Endothelial cells line cerebral capillaries and are linked to a vast network of pericytes and astrocyte end-feet, forming a blood-brain barrier (BBB). This structure selectively restricts the passage of substances from plasma into brain parenchyma. Dysfunction of the BBB is a potential APOE4-mediated pathway toward dementia. Indeed, loss of BBB integrity and subsequent vasogenic edema are common consequences of acute ischemia (Yang et al., 2019). Moreover, chronic activation and retraction of pericytes and endothelial cells may perpetuate pre-existing neurodegeneration in humans (Lau et al., 2020; Montagne et al., 2020a) and animal models (Bell et al., 2012). Various vascular risk factors are also associated with BBB damage (Cortes-Canteli and Iadecola, 2020). Dynamic contrast-enhanced magnetic resonance imaging (MRI) studies illustrate associations between APOE4 and limbic BBB breakdown in AD (Montagne et al., 2020a) and pericyte dysfunction in human stem cell-derived BBB models of CAA (Blanchard et al., 2020). Akin to atherosclerosis, mediation between APOE4 and dementia by the BBB may be unique to amyloid/tau response (Montagne et al., 2020a). Hence, VCID and AD likely share intermediate mechanisms, including ICAD and BBB dysfunction.

### Vascular Aging

Aging may contribute to vessel disease and VCID (Ungvari et al., 2018). The relationship between age and VCID is partially attributable to age-related reduction in cholesterol metabolism/clearance, promoting hypercholesterolemia, atherosclerosis, and cognitive changes (Wang and Bennett, 2012; Zlokovic et al., 2020). Additionally, aging may promote hypertension, vessel stiffness, and disease *via* lipid peroxidation, oxidative stress, mitochondrial dysfunction, and senescence of stroma, vasculature, and brain parenchyma (Gustaw-Rothberg et al., 2010; Tarantini et al., 2019; Kiss et al., 2020). These metabolic changes could be exacerbated by APOE4 (Yin et al., 2019).

## Potential Future Therapies

### Targeting Cholesterol in Cerebral Arteries: Statins and Atherosclerosis Therapy

We now address potential therapeutic targets for both atherosclerosis and dementia. Atherosclerosis management and research have overwhelmingly been guided by stroke outcomes rather than cognition. Nevertheless, because atherosclerosis is a strong risk factor for VCID, cholesterol optimization is a current treatment strategy for cerebrovascular diseases. Statins lower LDL by inhibiting hydroxymethyl glutaryl-CoA reductase, thus increasing LDL receptor expression through sterol-mediated regulatory response in the liver (Brown and Goldstein, 1986). Though statins are an essential treatment for ICAD, CAS, and stroke, the results for VCID and AD treatments are mixed. While robust randomized controlled trials had not yet been performed for VCID and statins, cohort studies imply that statins may significantly reduce the incidence of VCID in participants with vascular risk factors such as hypercholesterolemia or diabetes (Hajjar et al., 2002; Fei et al., 2013; Giannopoulos et al., 2014). Moreover, statins may mitigate cognitive progression in adults with normal cognition and MCI (Steenland et al., 2013). *APOE4* homozygotes emerged from re-analysis of statin trials with the greatest benefit, including slower cognitive decline and lower dementia incidence (Geifman et al., 2017). A meta-analysis found that statins may significantly lessen the risk of AD and MCI (Chu et al., 2018), and supporting studies trended toward benefit (Smith et al., 2017). However, numerous investigations report that statins do not prevent dementia while those studies that do support usage are often limited by smaller sample sizes (Shepardson et al., 2011a, b; McGuinness et al., 2016). Despite unclear evidence, the clinical co-occurrence of cerebrovascular disease and VCID often favors cholesterol management with statins.

To reduce infarcts that trigger/exacerbate VCID, current ICAD treatment includes cholesterol management, antiplatelet therapy (i.e., aspirin or P2Y_12_ inhibitors), and interventional methods (Chabriat and Bousser, 2006; Flusty et al., 2020). Yet, patients with ICAD on maximum medical therapy have non-negligible vascular risks, i.e., intracranial hemorrhage. One standard approach to treat cerebral ischemia with endovascular stenting even worsens outcomes significantly in ICAD (Chimowitz et al., 2011). Due to mixed findings, current neuro-interventional treatments are indicated for severe, symptomatic CAS only, but not for ICAD or VCID (Flusty et al., 2020). More research is required to better evaluate the possible benefit of ICAD treatment for VCID.

### Targeting Cholesterol in Glia and Macrophages: LXR Agonists and ACAT1 Blockade

Statins act mainly in the liver. Beyond statins, additional strategies that act in local tissues exist to modulate cholesterol metabolism in-principle. We cite two examples. Liver X Receptors (LXRs) are essential membrane receptors that regulate cholesterol efflux in macrophages (LXRα) as well as in astrocytes and glia (LXRβ). LXRs represent possible targets for ICAD and VCID. Indeed, activation of LXRs reduces serum cholesterol and increases ABCA1-mediated cholesterol transport in humans and mouse/primate models of atherosclerosis (Calkin and Tontonoz, 2011; Muse et al., 2018), mainly by modulating lipid load in macrophages (Zhang et al., 2014). Further, LXR agonist attenuates amyloid pathology and microglial inflammation in transgenic AD models (Zelcer et al., 2007). However, current LXR agonists also adversely upregulate fatty-acid and triglyceride syntheses in mice and humans (Kirchgessner et al., 2016; Muse et al., 2018). Therefore, clinical translation may require investigation of the net impact of these opposing effects.

CEs are storage forms of cholesterol. In foamy macrophages, like those found in atherosclerotic lesions, CEs accumulate by acyl-CoA:cholesterol acyltransferase (ACAT, aka sterol O-acyltransferase, SOAT) converting cholesterol to CEs, and by cholesterol hydrolases cleaving CEs back to cholesterol (Brown et al., 1980). ACAT1 and ACAT2 enzymes are encoded by *SOAT1* (Chang et al., 1993) and *SOAT2* genes (Anderson et al., 1998; Cases et al., 1998; Oelkers et al., 1998). Both enzymes are integral endoplasmic reticulum membrane proteins; both are allosterically activated by cholesterol or oxysterols, and act on various sterols and long-chain fatty-acyl-CoAs as substrates (reviewed in Rogers et al., 2015). In healthy humans, ACAT1 is ubiquitously expressed across peripheral and brain tissue, whereas ACAT2 is mostly expressed in enterocytes and hepatocytes (reviewed in Chang et al., 2009). Cell models show that unlike statins (which inhibit cholesterol efflux), ACAT1 blockade promotes cholesterol efflux *via* ABCA1 mediated lipid efflux (Yamauchi et al., 2004). Mouse studies portray that reduction in myeloid ACAT1 alleviates diet-induced atherosclerosis (Huang et al., 2016; Melton et al., 2019). In AD studies, ACAT inhibitors significantly reduced amyloid accumulation in cell culture (Puglielli et al., 2001) and mouse models (Hutter-Paier et al., 2004). ACAT1/*SOAT1* gene knockout in mice reduces amyloid (Bryleva et al., 2010) and tau burden (Shibuya et al., 2015a), by stimulating autophagy in microglia and neurons (Shibuya et al., 2014, 2015b). Additionally, ACAT1 inhibition rescues CE accumulation in human AD neurons (van der Kant et al., 2019) and human stem cell-derived microglia with *TREM2* gene ablation (Nugent et al., 2020). These studies suggest that ACAT1 is a potential target to address atherosclerosis and proteinopathy in VCID or mixed dementia. Moreover, in a mouse model for the Niemann-Pick type C disease, a neurological disease caused by primary genetic defects in cholesterol homeostasis, ACAT1 blockade diverts cholesterol from storage to promote more efficient utilization in neural and peripheral tissues, suggesting that targeting ACAT1 is a potential strategy for multiple neurological diseases that involve cholesterol dyshomeostasis (Chang et al., 2020). Humans ACAT1/*SOAT1* genetic variant analyses also support the role of ACAT1 in modulating dementia susceptibility (Wollmer et al., 2003; Alavez-Rubio et al., 2021).

### APOE-Based Therapy

Investigational dementia therapies include targeting APOE directly, such as reducing APOE4 expression, with antibodies (Xiong et al., 2021), anti-sense oligonucleotides (Huynh et al., 2017), gene therapy, and gene editing (Liu et al., 2013; Yamazaki et al., 2016). In fact, an individual harboring a frameshift mutation ablating *APOE* expression had relatively normal cognition (Mak et al., 2014), possibly indicating treatment safety in altering *APOE* expression. To this end, adenoviral APOE2 knock-in approaches have already been tested in mice and in non-human primates (Williams et al., 2020). Small-molecule approaches include inhibiting deleterious APOE functions and correcting aberrant APOE structure induced by ε4 polymorphisms and associated with elevated amyloid, tau, and lipids (Mahley and Huang, 2012; Wang et al., 2018). Although it is still unclear whether APOE4 raises dementia risk by a gain of toxic function and/or loss of protective function (Kim et al., 2009; Serrano-Pozo et al., 2021), APOE-based therapies might address both mechanisms at local and systemic levels.

## Conclusion

Neurodegeneration due to vascular etiologies is multifactorial and complex. Here, we describe a working model linking cholesterol load with atherosclerosis, APOE4, and VCID. At the cellular level, we posit that cholesterol load causes chronic neuroinflammation in microglia and astrocytes and triggers systemic inflammation in macrophages. Crosstalk between neuroinflammatory and systemic inflammatory signaling leads to stromal dysfunction. APOE4 may exacerbate local cellular cholesterol dyshomeostasis and stimulate glial/stromal inflammation, metabolic dysfunction, and BBB breakdown. At the macroscopic level, these changes promote atherosclerosis, ischemia, and cerebrovascular disease, ultimately contributing to VCID. Moreover, vascular changes, cholesterol accumulation, inflammation, and impaired turnover of protein and/or lipid aggregates are shared across neurodegeneration. We review potential therapies targeting cholesterol metabolism in astrocytes, microglia, and macrophages, though our understanding is far from complete. Future research may delineate VCID pathophysiology towards effective treatment.

## Author Contributions

MD, T-YC, and CC conceived the project. MD wrote the initial version of the review and received inputs from IN, DW, CC, and T-YC for revisions. All authors contributed to the article and approved the submitted version.

## Conflict of Interest

The authors declare that the research was conducted in the absence of any commercial or financial relationships that could be construed as a potential conflict of interest.

## Supplementary Material

SUPPLEMENTARY FIGURE 1 Anatomy of cerebral arteries, including the (**A,B**) carotid arteries and (**C,D**) Circle of Willis (CoW), also shown on computed tomography arteriography (**B,D**). Illustrations adapted from Henry Gray’s *Anatomy of the Human Body* (1918), www.bartleby.com.
